# Effectiveness of Health-Led Cognitive Behavioral-Based Group Therapy on Pain, Functional Disability and Psychological Outcomes among Knee Osteoarthritis Patients in Malaysia

**DOI:** 10.3390/ijerph17176179

**Published:** 2020-08-26

**Authors:** Chai Nien Foo, Manohar Arumugam, Rampal Lekhraj, Munn-Sann Lye, Sherina Mohd-Sidik, Zubaidah Jamil Osman

**Affiliations:** 1 Department of Population Medicine, Faculty of Medicine and Health Sciences, Universiti Tunku Abdul Rahman, Kajang 43000, Malaysia; lyems@utar.edu.my; 2 Department of Community Health, Faculty of Medicine and Health Sciences, Universiti Putra Malaysia, Serdang 43400, Malaysia; dr_rampal1@hotmail.com; 3 Department of Orthopaedic, Faculty of Medicine and Health Sciences, Universiti Putra Malaysia, Serdang 43400, Malaysia; 4 Department of Psychiatry, Faculty of Medicine and Health Sciences, Universiti Putra Malaysia, Serdang 43400, Malaysia; sherina@upm.edu.my; 5 Faculty of Allied Health Sciences, Cyberjaya University College of Medical Sciences, Cyberjaya 63000, Malaysia; zujamil@gmail.com

**Keywords:** knee osteoarthritis, patient education, pain, psychological outcomes, Malaysia

## Abstract

Background: Psychosocial interventions for patients with osteoarthritis (OA) of the knee to reduce pain and improve physical and psychological functioning are still lacking in Malaysia. Methods: A parallel-group unblinded randomized controlled trial involving 300 patients was conducted in two hospital orthopedics clinics in Malaysia. Patients were randomly assigned to receive cognitive behavioral-based group therapy (*n* = 150) or no further intervention (*n* = 150). The primary outcome was the change from baseline in knee pain as determined by the Knee injury and Osteoarthritis Outcome Score (KOOS) at 6 months. The data collected were analyzed by covariate-adjusted mixed design repeated measures analysis of variance. All analyses were performed under the terms of intention-to-treat. Results: At 6 months, mean change from baseline in the KOOS knee pain score was 0.6 points (95% CI −1.73 to 2.94) in the control group and 8.9 points (95% CI 6.62 to 11.23) (denoting less knee pain intensity) in the intervention group (significant treatment effect *p* < 0.0001). Patients treated with such an approach also experienced significant improvement in functional ability when performing activities of daily living and had improved ability to cope with depression, anxiety and pain catastrophizing. Conclusion: The intervention module delivered by healthcare professionals had a sustained effect on knee OA pain and functionality over 6 months, thereby leading to an overall improvement in psychological well-being, thus benefitting most of the Malaysian knee OA patients.

## 1. Introduction

Osteoarthritis (OA) is a highly prevalent chronic disabling joint disease that affects one in eight adults globally [1]. It mainly affects the knee joint [2], causing pain and functional disability [3]. It is estimated that 85% of the total OA burden is knee OA [4]. The years lived with disability (YLDs) rate due to knee OA increased by 35% from 2005 to 2015 [4]. Knee OA is a huge burden on the patient. Conventional interventions fail to treat OA completely while surgical interventions such as total hip replacement (THR) or total knee replacement (TKR) may lead to problems such as infection and mechanical complications including reduced mobility [5,6]. According to Jensen and colleagues (2014), current available intervention for knee OA including medical, pharmaceutical, and surgical intervention for chronic OA pain are inadequate and moderately effective at best. Hence, the authors suggested alternative therapy to be considered when patients knee pain does not improve after conventional intervention [7]. Although advanced approaches such as regenerative medicine and stem cell therapy have been studied in managing various musculoskeletal disorders including osteoarthritis, all these interventions do not sufficiently address the psychological well-being relating to knee OA [8,9].

While OA affects patients bio-psychosocially, most seek only intervention to treat their physical condition while only a limited number seek psychosocial support such as with cognitive behavioral therapy (CBT). As chronic pain secondary to OA can also be caused by psychological factors, it is crucial to address the psychological aspect that includes the emotional, cognitive and behavioral outcomes contributing to OA knee pain [10]. However, such an approach is not typically integrated into primary and medical specialty practices owing to insufficient clinical psychologists in CBT in comparison to the number of knee OA patients in Malaysia. CBT is a popular therapeutic approach to treating a wide range of disorders; its goal is to change the patterns of thinking behavior and attitude underlying the disorders and pathologies [11]. In Malaysia, CBT has been evaluated for its effectiveness in treating type-2 diabetes [12], depression [13], and chronic pain [14].

Despite the importance of CBT in chronic pain, there has been very little research on evaluating psychosocial interventions for knee OA patients. In fact, there is no psychosocial intervention in Malaysia for patients with OA of the knee focusing primarily on reducing pain and improving physical and psychological functioning. Owing to insufficient clinical psychologists, physiotherapists and nurses who are usually the ones handling patients with knee OA should be in the forefront of health-led psychosocial interventions in promoting pain self-management, which have become increasingly popular in primary care and hospitals [15].

The objective of this study is to develop a cognitive behavioral-based therapy intervention module for physiotherapists and nurses and to evaluate its effectiveness in treating pain, functional disability and the psychological outcomes of knee OA patients in Malaysian tertiary hospitals.

## 2. Materials and Methods

### 2.1. Study Design and Site

This study was a two-arm parallel group randomized clinical trial involving diagnosed knee OA patients who fulfilled the criteria as participants for the study that was conducted. The patients were recruited from Orthopedics Clinics in Hospital Putrajaya and Hospital Serdang, both of which were located in the Greater Kuala Lumpur and Klang Valley (Greater KL/KV) region, Malaysia.

### 2.2. Sample Size

The sample size, *n*, was determined following the calculation for hypothesis testing by comparing means, where *n* = 2δ^2^(Z_1−α/2_ + Z_1−β_)^2^/(µ_1__−_ µ_2_)^2^ [16]. To detect a 20% increase in mean baseline knee pain score from baseline values over a 6-month period [17] and power of 80% assuming a two-tailed test with a type 1 error rate of 5%, this study recruited 300 participants allowing for a 20% attrition [18].

### 2.3. Participants

The participants comprised 300 patients aged between 35 and 75 years who agreed to be in the study. They had been diagnosed with primary knee OA on the basis of medical evaluation (knee pain for most days of the month before and bony enlargement of the knee) and radiographic examination showing Kellgren–Lawrence (K-L) classification of grade 2 or higher. They also had an average pain intensity of 40 or higher on a 100 mm visual analogue scale in the 7 days before baseline assessment. Patients excluded from this study were those with any of the following conditions: knee pain caused by conditions other than knee OA; had knee replacement surgery of the affected knee in the past year; were currently receiving or had undergone cognitive behavioral-based therapy or other psychotherapy (including counseling); had participated in any other clinical study in the past 12 months; had been diagnosed with mental disorder, pregnancy or were breastfeeding.

### 2.4. Randomization and Blinding Procedure

After written informed consent was obtained from the selected patients, they were randomly allocated to either the intervention or control group based on a block randomization method that used an independently operated computer-generated random sequence system (http://random-allocation-software.software.informer.com/2.0/). To ensure similar treatment numbers for each group, blocked randomization of six was used in this study. At that stage, the treatment allocation was concealed from the following: the participants, the researcher involved in recruitment, hospital staff and therapists.

Because of the nature of the intervention, blinding could not be applied to the participants, the cognitive behavioral-based therapy therapists or the researchers. However, research assistants who assessed the outcomes were blinded to assigned grouping.

### 2.5. Intervention

The education module in this study adopted the cognitive behavioral model in its framework. It emphasized behavioral activation and resetting the perception and negative thoughts related to knee pain, unlike standard CBT treatments focused primarily on coping, and it did not directly address physical impairments. Participants in both intervention and control groups received standard routine care throughout the study. They attended clinic and physiotherapy sessions as usual on their fixed appointment dates. All participants received advice on symptom management and standard exercises to remain active. The participants in the “passive” control group received no further intervention and were each provided with The Knee Book. Participants in the intervention group, besides being given The Knee Book, also received three sessions of group cognitive behavioral-based therapy. The two-and-a-half-hour sessions were conducted bi-weekly in groups of eight to twelve participants.

Each session began with an introduction and lecture, a problem-solving task, skills training, homework assignments and feedback of the session which took approximately 45 min to complete. A meal was provided after the lecture to enhance peer support and social bonding during the 15-min break. The session ended with exercises, diaphragmatic breathing, knee muscle relaxation and a six-minute walk test. In the first session, a lecture was given to serve as an ice breaker and to provide an overview of the program. Information on chronic pain and goal setting was also covered. The second session introduced time-based pacing, while the final session included lectures on good quality sleep, relapse prevention and dealing with flare-ups. Compliance with cognitive behavioral-based therapy intervention program was defined as attendance at all three sessions.

### 2.6. Training of Health-Led Therapists

There were two physiotherapists and two nurses who were trained by a senior clinical psychologist to deliver the intervention program. An advantage of having physiotherapists or nurses deliver the intervention program was that they could integrate exercises and psychosocial treatment that applied the cognitive behavioral approach in this intervention program into a single session. It also increased accessibility of the program to individuals who might not otherwise have access to a clinical psychologist. Moreover, this arrangement reduced overall health care costs.

To ensure that the physiotherapists and nurses selected had adequate experience and knowledge in the management of knee OA, only those who had working experience in either the rehabilitation unit or the orthopedics clinic for more than one year were invited to participate in the study. They received at least one day’s training specific to the trial from an experienced senior clinical psychologist with cognitive behavioral therapy experience and practiced role play sessions with an orthopedic specialist. They were accredited to deliver the intervention program after audio-recordings of each practice session with a group of knee OA patients were reviewed by the clinical psychologist to ensure that the criteria for content and quality of delivery were met.

Therapists’ competence in the delivery of intervention was assessed for a random selection of sessions by two experienced clinical psychologists with the Revised Cognitive Therapy Scale (CTS-R) [19], which is a valid and reliable CBT rating scale. To ensure the intervention’s quality, the raters were not informed about the stage of intervention at which the recordings were made.

### 2.7. Outcome Measures

The primary outcome variable of this study was knee pain intensity. Secondary outcome measures of this study were functional disability (daily living and sports) and various psychological outcome measurements that included depression, anxiety, stress, fear-avoidance beliefs (physical activity and work), pain catastrophizing and pain self-efficacy. Outcome variables were compared at baseline, and the effects of intervention on changes in outcome measures were determined immediately after intervention and one month and six months post treatment. The primary efficacy endpoint was the mean change in Knee injury and Osteoarthritis Outcome Score (KOOS) knee pain score against baseline over a period of six months.

Data for the intervention outcomes were recorded for the KOOS knee pain score [20], the 21-item Depression Anxiety and Stress Scale (DASS-21) [21], Fear-avoidance Beliefs Questionnaire (FABQ) [22], Pain-related Self Statements (PRSS) [23] and Pain Self Efficacy Questionnaire (PSEQ) [24]. This study adopted, with permission from authors, the pre-tested and validated English and Malay version questionnaires.

### 2.8. Statistical Analysis

The data collected were analyzed using descriptive and inferential statistics with the application of Statistical Package for Social Sciences software (SPSS) version 21. Before the principal data analysis was performed, exploratory data analysis (EDA) was undertaken to detect any outliers, missing values, test for normality, equality of variance and multicollinearity. Analysis of outcomes was by intention-to-treat where all participants who were randomized and entered the trial were included in the analysis in the condition to which they were assigned without imputation of missing data to avoid selection bias. Mixed design repeated measures ANOVA was applied to determine the effects on mean scores of the outcomes in both intervention and control groups. The treatment effects were adjusted for age, gender and body mass index. A *p* value of less than 0.05 was taken as statistically significant.

### 2.9. Ethical Approval

Prior to the enrolment of participants, approval for the protocol of this study was obtained from the National Medical Research Register (NMRR), Ministry of Health Malaysia (NMRR-15-74-24008) and Universiti Putra Malaysia Ethics Committee (UPM/TNCPI/RMC/1.4.18.1 (JKEUPM)/F2) for research involving human subjects.

## 3. Results

### 3.1. Characteristics of Participants

**Figure 1:**
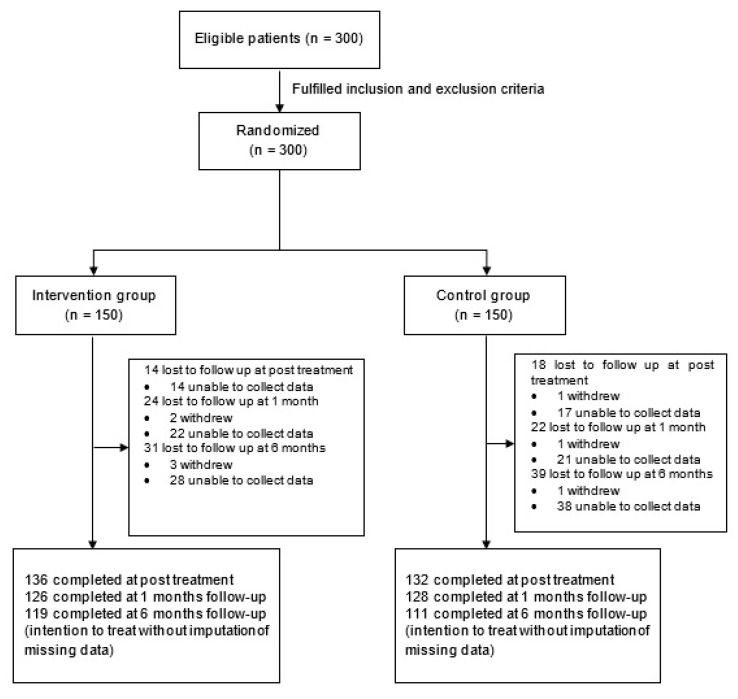
Consort flow chart of intervention and control groups.

Two hundred and thirty of the 300 randomized participants completed the six month follow up assessment and were included in the analysis, giving a response rate of 76.67% (Figure 1). Detailed baseline socio-demographic characteristics of the 300 participants had been published elsewhere [25]. The majority of the participants were women (82.7%), aged 56–65 years (38.0%) and were Malay (64.0%). One hundred and seventy eight (59.3%) participants were diagnosed with unilateral knee OA. The overall mean age for the participants first diagnosed with knee OA was 50.10 years (SD = 10.04, 95% CI 48.96 to 51.24). Two hundred and eighteen (72.7%) participants had body mass index less than 30. The majority (43.3%) were diagnosed with K-L grade II knee OA and 35.3% with K-L grade III knee OA. Only a small number (21.3%) were diagnosed with K-L grade IV knee OA.

The majority (68.9%) of the participants had knee pain score of four to six, and the remainder (31.1%) had knee pain score of seven and above according to the 100 mm visual analogue scale. The overall mean score for knee pain was 5.59 (SD = 1.623, 95% CI 5.59 to 5.97).

Of the 150 participants who began the intervention, 12 did not complete all three sessions but participated in at least two sessions of the intervention program. The compliance rate of participants in the intervention group was therefore 92%. Those 12 participants who did not comply with cognitive behavioral-based therapy intervention were included in the “intention-to-treat” analysis. All the sessions’ content was delivered as intended and followed the prespecified criteria of good practice of CBT.

### 3.2. Effectiveness of Intervention on Outcome Measures

Table 1 shows the effectiveness of cognitive behavioral-based therapy at the intervention and at one month and six months post intervention. The mean KOOS knee pain score increased by 0.6 points (95% CI −1.73 to 2.94), which was not significantly different between baseline and after 6 months in the control group. On the other hand, the KOOS pain score rose (signifying lower knee pain intensity) by 8.9 points (95% CI 6.62 to 11.23) in the intervention group. This moderate difference represented a significant treatment effect (*p* < 0.0001, partial η^2^ = 0.12), indicating that the intervention had resulted in a measurable degree of knee pain relief among participants.

The mean KOOS functional disability (daily living) score increased significantly by 4.82 points (95% CI 1.49 to 8.15) from baseline to 6 months in the control group. The intervention group recorded an increase in 9.95 points (95% CI 6.65 to 13.24) signifying lower symptoms of functional disability. This was a significant treatment effect (*p* < 0.0001, partial η^2^ = 0.036). This small effect size indicated that a detectable improvement in KOOS functional disability (daily living) occurred among participants following the intervention.

The mean KOOS functional disability (sport) score in the control group increased by 2.82 points (95% CI −4.86 to 10.50) at six months, this change not being significantly different from baseline. The corresponding reading in the intervention group increased by 5.45 points (95% CI −2.14 to 13.04), this change not being significant either (*p* = 0.681, partial η^2^ = 0.02).

The mean depression score that decreased by 0.01 points (95% CI −0.91 to 0.89) in the control group was not significantly different between baseline and 6 months. On the other hand, the decrease of 2.17 points (95% CI −3.06 to −1.28) in the intervention group was statistically significant (*p* < 0.0001, partial η^2^ = 0.083). This moderate effect size indicated that implementation of the intervention resulted in a measurable improvement in depression among the participants.

The mean anxiety score in the control group decreased by 0.19 points (95% CI −1.64 to 1.26), a change that was not significantly different from baseline to 6 months. The corresponding decrease in the intervention group was 1.83 points (95% CI −3.26 to −0.39), representing a significant treatment effect (*p* = 0.006, partial η^2^ = 0.021). This small effect size indicated that an improvement in anxiety among participants was detectable following intervention.

In the control group, the mean stress score decreased by 0.40 points (95% CI −1.84 to 1.05), the difference not being significantly different from baseline after 6 months. A similar trend was observed in the intervention group where the decrease of 0.33 points (95% CI −1.76 to 1.10) was also not significant (*p* = 0.552, partial η^2^ = 0.003).

For the mean fear-avoidance beliefs (work) score, the control group registered a decrease of 2.57 points (95% CI −5.98 to 0.86) that was not significantly different from baseline to 6 months. The intervention group did not fare better, with a non-significant lowering by 2.05 points (95% CI to −5.43 to 1.33) (*p* = 0.956, partial η^2^ = 0.0001).

The mean fear-avoidance beliefs (physical activity) score decreased non-significantly by 2.09 points (95% CI −4.39 to 0.21) for the six month duration from baseline. The same tendency was true for the intervention group which registered scores that showed a mean decrease of 1.54 points (95% CI −3.81 to 0.73). Here again, there was no significant treatment effect (*p* = 0.838, partial η^2^ = 0.001).

The mean pain catastrophizing score in the control group decreased by 0.23 points (95% CI −0.56 to 0.09); this was not a significant shift from baseline after 6 months, whereas in the intervention group, there was significant decline of 0.88 points (95% CI −1.20 to −0.56). For the latter, the treatment effect was significant (*p* < 0.0001, partial η^2^ = 0.052). This small effect size indicated that implementation of the intervention resulted in a detectable improvement in pain catastrophizing among participants.

The mean pain self-efficacy score increased not significantly by 0.26 points (95% CI −0.16 to 0.69) from baseline to 6 months in the control group. The change in the intervention group was significantly increased by 0.54 points (95% CI 0.12 to 0.96). However, there was no significant treatment effect (*p* = 0.253, partial η^2^ = 0.006).

## 4. Discussion

Effective intervention can result in the reduction of osteoarthritic pain leading to improvement in quality of life [26]. This study examined the cognitive behavioral-based therapy module implemented by nurses and physiotherapists in managing knee osteoarthritic patients. The effectiveness of the approach was evaluated based on pain, functional disability during activities of daily living and sports, depression, anxiety, stress, fear-avoidance beliefs, pain catastrophizing and pain self-efficacy. The findings showed that patients treated with such an approach experienced significant relief in knee pain, improvement in functional ability when performing activities of daily living and had improved ability to cope with depression and anxiety as well as better response to pain catastrophizing.

Pain is associated with many health conditions, disturbed functions and reduced activity in daily living and is a common reason for seeking medical attention [27]. In osteoarthritic individuals, pain is a well-known contributing factor that reduces the quality of life, leading to substantial socioeconomic burden [28,29,30,31,32]. Hence, addressing pain may improve the prognosis and quality of life of osteoarthritic patients and help in the reduction of their socioeconomic burden. The present study shows that integration of cognitive behavioral-based therapy into current therapy significantly improved pain outcomes of knee osteoarthritic patients. The findings of this study are consistent with a meta-analysis of the effect of arthritis self-management education programs on pain and disability [33]. Although current therapies focusing on the physiological aspect of the condition such as exercise therapy, kinesiotaping, hydrotherapy and drugs such as etoricoxib have been successful in relieving pain [34,35,36,37], integrating cognitive behavioral-based therapy into these approaches may further enhance their effectiveness via the psychological dimension.

Functional ability in performing activities of daily living and sports has been associated with various physio-medical conditions such as body alignment, presence of deformity and pain [38,39,40,41]. In the osteoarthritic knee, pain and deformity are among the main contributing factors to the reduction of functional ability [42,43,44]. The present findings showed that integrating cognitive behavioral-based therapy into intervention resulted in significant improvement in functional ability when performing activities of daily living. Nevertheless, the improvement in functional ability in sports was not found to be significant, presumably because sporting activities require a certain level of training and practice [45,46,47].

Osteoarthritis also affects the individual’s psychosocial wellbeing expressed through depression, anxiety and stress [48]. Hence, addressing the problems associated with osteoarthritis may restore such patients back to a level of psychological fitness which indirectly improves their social health. Our findings demonstrate that incorporation of cognitive behavioral-based therapy delivered by physiotherapists and nurses resulted in a significant reduction in depression and anxiety. These observed effects are similar to the findings in a clinical study of an internet-delivered CBT for managing chronic pain where a large effect was identified for both depression and anxiety [49].

Results from the present study did not show significant improvement in stress. Nevertheless, stress is a broad general term to describe the response towards pressure, overwork or fatigue, besides being a result of external factors such discomfort from osteoarthritis [50]. Therefore, more time might be required to rehabilitate the patient under these circumstances.

Our study also found no significant improvement in fear avoidance beliefs and pain self-efficacy. Fear avoidance is the avoidance of a certain activity due to fear of increased pain [51], while pain self-efficacy is the confidence in one’s ability to perform functional activity effectively while in pain [52]. Both are important elements in rehabilitating patients to enable ease in functions of daily living. However, we believe that these traits require frequent practice in certain activities to enable patients to regain confidence and overcome their fear avoidance beliefs [53]. Besides, the fact that the intervention program module in the present study includes only a six-minute walk test, rather than incorporating other physical performance tests including stair climb, lifting carrying task, standing balance and chair stands, might partly explain the non-significant findings in fear-avoidance beliefs.

On the other hand, we observed that incorporating CBT into physiotherapy and nursing intervention brought about significant improvement in pain catastrophizing, which is defined as helplessness, active rumination and over-magnification of cognitions and feelings when dealing with an actual or anticipated painful condition [54]. These findings were comparable to the short-term effects of the internet-delivered CBT for managing chronic pain reported by Dear et al. (2013) [49]. Besides conventional therapies such as physiotherapy and nursing intervention for OA knee pain patients, other interventions such as emotional freedom techniques (EFT) are also alternatives reported to be effective in managing pain arising from fibromyalgia and frozen shoulder [55,56]. Hence, future studies on incorporating CBT into EFT should be carried out to compare its effectiveness to that of mainstream therapy in order to provide OA patients with a wide choice of effective interventions.

## 5. Conclusions

Incorporation of cognitive behavioral-based therapy into physiotherapy and nursing management yielded many positive outcomes in osteoarthritis rehabilitation. The involvement of physiotherapy and the role of nurses in knee osteoarthritic patient care may overcome the problem of the lack of clinical psychologists to advise patients in self-managing their own conditions. In view of the various positive effects observed so far, future studies should explore incorporating different types of physiotherapy interventions to determine the best combination for the management of knee OA.

### Strengths and Limitations

The randomized controlled trial study design and adequate sample size are the strengths of this study, allowing generalizability to knee OA patients in Malaysia. Standard routine care for the control treatment applied in this study is common for the primary care of OA to allow indirect comparison across trials [57].

A potential limitation of this study is that the sample consisted of 17% of male participants. As such, the treatment effects were adjusted for gender. Another limitation is the possibility of optimal session of cognitive behavioral-based group therapy to achieve treatment effects. Future studies should explore dose comparisons of short with more intense of sessions for cognitive behavioral-based group therapy protocol. The short follow-up period of six months is a potential limitation to the assessment of the effectiveness of the psychological intervention effect. Modification of behavior in performing sports and overcoming fear-avoidance beliefs may require longer observation. Besides, it is necessary to study whether the intervention has a positive impact on performance-based lower extremity functional tests. This implies that lower extremity function is influenced by knee pain. Thus, the possibility that performance outcome of lower extremity function affects physical performance can be determined.

## Figures and Tables

**Table 1:** Effectiveness at immediate, 1 and 6 months post intervention.

TimePoint	Mean Change from Baseline (95% CI)		*p* Value (Interaction)	Partial Eta Square
	Control (*n* = 111)	Intervention (*n* = 119)		
**KOOS Knee Pain (points)**			<0.0001 *	0.122
Immediate	−0.190 (−1.108 to 0.728)	3.067 (2.159 to 3.974) *		
1 month	0.432 (−1.901 to 2.765)	4.264 (1.958 to 6.570) *		
6 months	0.603 (−1.732 to 2.938)	8.926 (6.618 to 11.234) *		
**KOOS Functional Disability (Daily Living) (points)**			<0.0001 *	0.036
Immediate	1.168 (−0.244 to 2.579)	1.464 (0.069 to 2.859) *		
1 month	2.459 (0.049 to 4.869) *	4.608 (2.225 to 6.990) *		
6 months	4.819 (1.485 to 8.153) *	9.948 (6.653 to 13.243) *		
**KOOS Functional Disability (Sport) (points)**			0.681	0.002
Immediate	2.419 (−3.006 to 7.845)	3.154 (−2.209 to 8.516)		
1 month	4.314 (−2.775 to 11.402)	5.292 (−1.714 to 12.298)		
6 months	2.821 (−4.856 to 10.497)	5.452 (−2.135 to 13.039)		
**Depression (points)**			<0.0001 *	0.083
Immediate	−0.004 (−0.655 to 0.648)	−0.064 (−0.708 to 0.580)		
1 month	−0.330 (−1.088 to 0.428)	−0.807 (−1.556 to −0.057) *		
6 months	−0.008 (−0.909 to 0.893)	−2.167 (−3.057 to −1.276) *		
**Anxiety** **(points)**			0.006 *	0.021
Immediate	−0.019 (−1.054 to 1.016)	−0.382 (−1.405 to 0.641)		
1 month	0.273 (−0.917 to 1.464)	−0.932 (−2.108 to 0.245)		
6 months	−0.189 (−1.640 to 1.261)	−1.827 (−3.260 to −0.394) *		
**Stress** **(points)**			0.552	0.003
Immediate	0.259 (−0.454 to 0.972)	0.015 (−0.690 to 0.719)		
1 month	0.134 (−0.974 to 1.242)	−0.369 (−1.464 to 0.726)		
6 months	−0.397 (−1.844 to 1.049)	−0.326 (−1.756 to 1.103)		
**Fear-Avoidance Beliefs (Work) (points)**			0.956	0.000
Immediate	−0.720 (−3.934 to 2.495)	−0.462 (−3.640 to 2.715)		
1 month	−1.986 (−5.203 to 1.230)	−1.798 (−4.977 to 1.381)		
6 months	−2.565 (−5.984 to 0.855)	−2.053 (−5.433 to 1.326)		
**Fear-Avoidance Beliefs (Physical Activity) (points)**			0.838	0.001
Immediate	−0.450 (−1.950 to 1.050)	−0.423 (−1.906 to 1.059)		
1 month	−1.126 (−3.212 to 0.959)	−0.829 (−2.890 to 1.232)		
6 months	−2.094 (−4.393 to 0.205)	−1.538 (−3.811 to 0.734)		
**Pain Catastrophizing (points)**			<0.0001 *	0.052
Immediate	0.058 (−0.121 to 0.237)	−0.083 (−0.260 to 0.094)		
1 month	−0.108 (−0.388 to 0.172)	−0.517 (−0.794 to −0.240) *		
6 months	−0.234 (−0.555 to 0.087)	−0.880 (−1.197 to −0.563) *		
**Pain Self-Efficacy (points)**			0.253	0.006
Immediate	0.019 (−0.173 to 0.210)	0.183 (−0.007 to 0.372)		
1 month	0.129 (−0.194 to 0.453)	0.235 (−0.085 to 0.555)		
6 months	0.264 (−0.160 to 0.688)	0.543 (0.123 to 0.962) *		

Knee injury and Osteoarthritis Outcome Score (KOOS) knee pain score increased indicates lower symptoms of pain. KOOS function (daily living and sport and recreation) score increased indicates lower symptoms of functional disability (daily living and sport and recreation). Depression score increased indicates higher depression level. Anxiety score increased indicates higher anxiety level. Stress score increased indicates higher stress level. Fear-avoidance beliefs (work and physical activity) score increased indicates higher fear-avoidance beliefs (work and physical activity) level. Pain catastrophizing score increased indicates higher pain catastrophizing level. Pain self-efficacy score increased indicates higher pain self-efficacy level. Interaction (group x time) represents the treatment effect as the difference in change from baseline between the two groups. * Significant at *p* < 0.05.
